# Unveiling recent and ongoing adaptive selection in human populations

**DOI:** 10.1371/journal.pbio.3002469

**Published:** 2024-01-18

**Authors:** Ziyue Gao

**Affiliations:** Department of Genetics, Perelman School of Medicine, University of Pennsylvania, Philadelphia, Pennsylvania, United States of America

## Abstract

Genome-wide scans for signals of selection have become a routine part of the analysis of population genomic variation datasets and have resulted in compelling evidence of selection during recent human evolution. This Essay spotlights methodological innovations that have enabled the detection of selection over very recent timescales, even in contemporary human populations. By harnessing large-scale genomic and phenotypic datasets, these new methods use different strategies to uncover connections between genotype, phenotype, and fitness. This Essay outlines the rationale and key findings of each strategy, discusses challenges in interpretation, and describes opportunities to improve detection and understanding of ongoing selection in human populations.

## Abstract 2

What is the evidence for ongoing selection in recent human evolution? This Essay looks at how new methods and techniques are helping to uncover connections between genotype, phenotype, and fitness at a population level.

## Introduction

**Fig 1:**
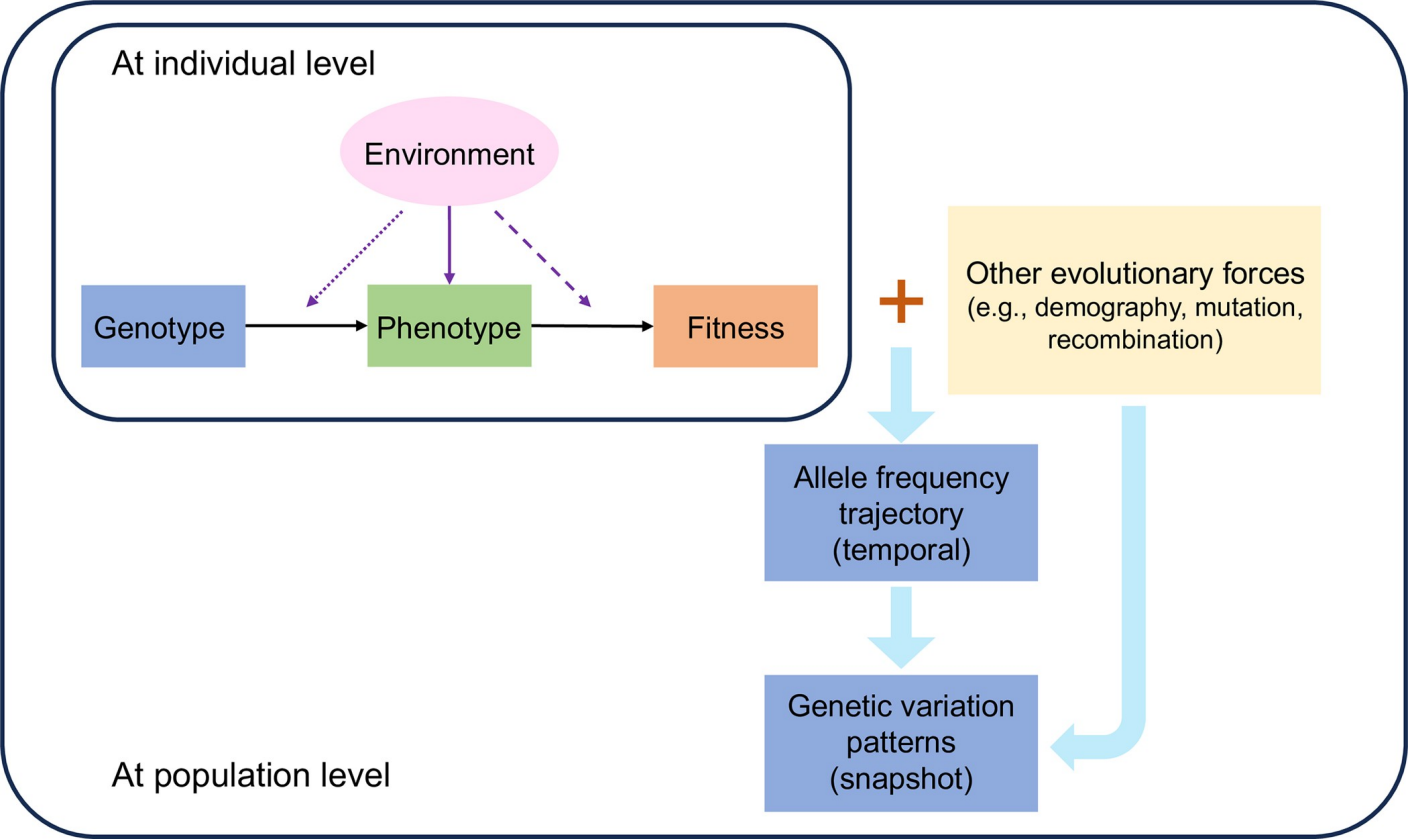
Overall framework of selection bridging population genetics and quantitative genetics models. In this conceptual framework, selection on genotype is mediated by fitness-relevant phenotype and manifests in allele frequency changes and genetic variation patterns. In any specific environment, genotype and environment together shape the phenotype of an individual, which in turn determines the fitness. In addition to its direct effect on the phenotype (solid purple arrow), the environment also modifies the genotype-to-phenotype mapping (i.e., genotype-by-environment interaction; indicated by the dotted purple arrow) and phenotype-to-fitness mapping (dashed purple arrow). Through interactions with other evolutionary forces (indicated by the brown plus sign), natural selection shapes the allele frequency trajectory over time and leaves footprints in genomic variation in present-day populations.

**Fig 2:**
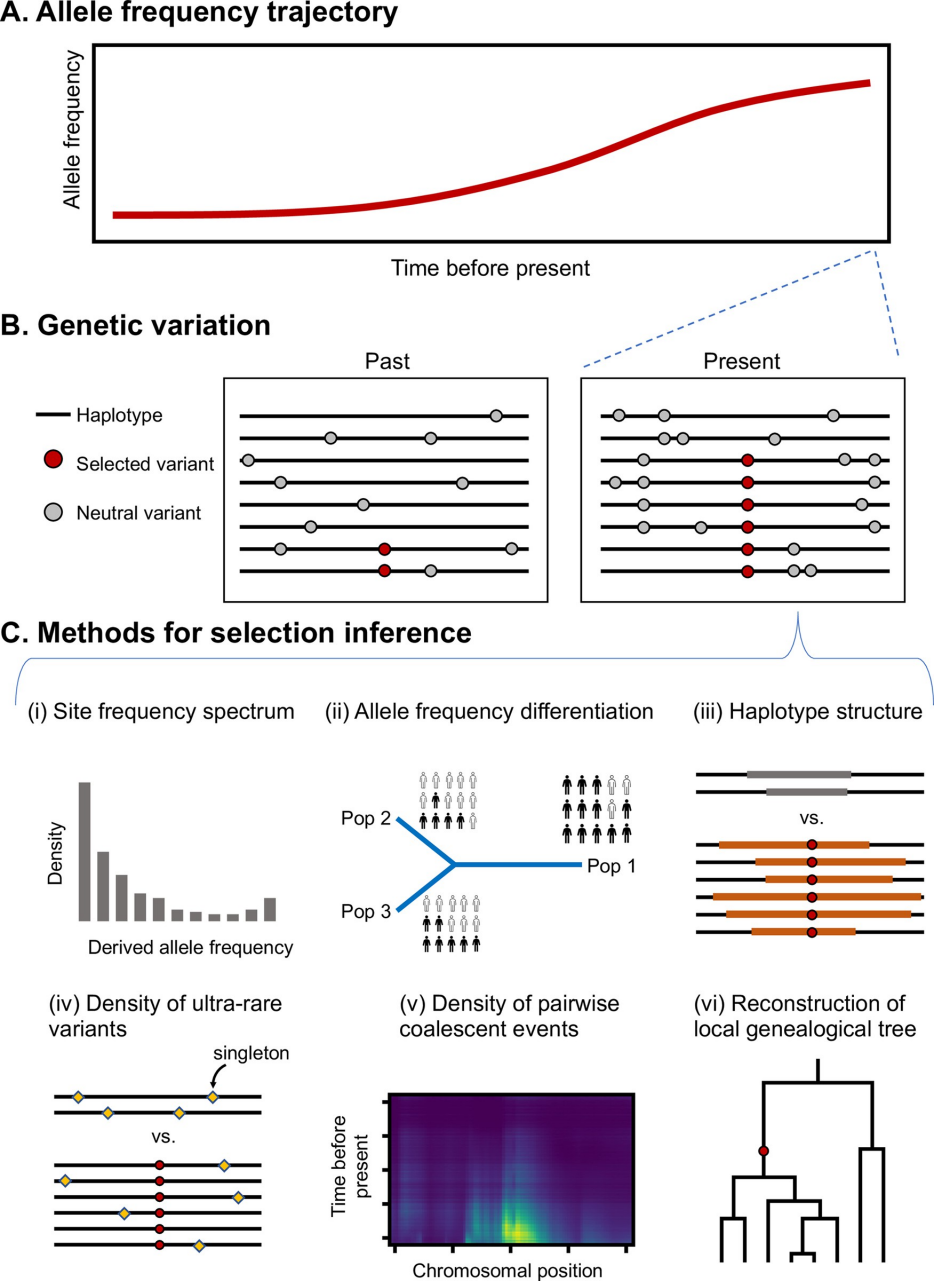
Signals of recent positive selection in genetic variation and corresponding methods for selection inference. (A) The hallmark of positive selection is faster allele frequency increase than would be expected under neutrality. (B) The rapid allele frequency change leaves footprints in the surrounding genomic region, although the specific patterns depend on the strength, tempo, and mode of selection (e.g., selection on standing variation versus on de novo variants). (C) Major methods for detecting positive selection based on present-day genetic variation.

A central query in human evolutionary genetics is to understand the functions and evolutionary history of genes or genomic regions that are under natural selection. Selection favors genetic variants that lead to advantageous phenotypic changes in specific environments, resulting in increases in allele frequency over time and distinctive patterns of genetic variation in present-day populations (Figs 1, 2A and 2B). Beyond unraveling the origin and evolutionary history of these selective genetic changes, it is of immense interest to gauge their contribution to phenotypic diversity in present-day human populations, as well as their impacts on disease risk and overall fitness (Box 1) in contemporary environments. Therefore, recent research endeavors are increasingly shifted towards identifying and characterizing extremely recent and even ongoing selection.

**Box 1. Glossary**

**Fitness**

A measure of how well an individual can survive or reproduce; it consists of multiple components such as viability, mating success, and fecundity.

**Positive selection**

An evolutionary process in which a genetic variant becomes more common in a population because it increases the fitness of individuals who carry it.

**Negative selection**

An evolutionary process that weeds out fitness-reducing genetic variants from the population. Purifying selection acts directly on the deleterious variants, whereas background selection affects nearby variants linked to the deleterious variants.

**Positive and negative selection**

Two inseparable concepts that describe the same phenomenon from different angles. To facilitate communication, population geneticists often adopt either of these terms focusing on the impact of selection on the derived allele, such that positive selection tends to speed up molecular evolution, whereas negative selection decelerates or prevents it. Nonetheless, in many cases, identity of the derived allele is ambiguous or less relevant (e.g., during transient selection), and the direction of selection often refers to the effect of selection on the rare allele (for example, a scenario where the rare allele is beneficial is often considered positive selection, although one could consider the same scenario as negative selection against the more common allele).

**Genetic adaptation**

The process by which organisms evolve heritable characteristics or traits that help them to better survive and reproduce in their specific environment. In many cases, adaptation is used synonymously with positive selection, but adaptation also encompasses other selection modes such as balancing selection and polygenic adaptation.

**Stabilizing selection**

A type of natural selection that favors individuals with an intermediate value of a fitness-relevant trait. Individuals with deviation from the optimal trait value are selected against, and the result is a stabilization of the trait around a specific value. Stabilizing selection concerns the relationship between phenotype and fitness, regardless of the genetic basis. Other types of phenotype-focused selection include disruptive selection, which favors individuals with extreme trait values, and directional selection, which favors individuals at only one end of the phenotypic spectrum.

**Polygenicity**

Polygenicity refers to a scenario in which variation in a trait within a population is contributed to by genetic variants at multiple genes or genomic loci rather than by just one or a few. Many complex traits in humans, such as height and disease susceptibility, are highly polygenic.

**Pleiotropy**

Pleiotropy occurs when a single genetic variant (or gene) influences two or more seemingly unrelated phenotypes in an organism. Two traits are pleiotropically related when certain variants exist that simultaneously affect them.

Numerous scans have been carried out in the human genome for targets under selection of intermediate scales (e.g., over 1,000 generations), but it remains a challenging task to demonstrate that selection on the identified targets is still ongoing or to detect selection that started recently. Enabled by the recent availability of population-scale genomic data and the development of efficient algorithms for inferring local genealogical trees, many new methods have been developed in the past 20 years to detect signals of selection from the past few millennia (e.g., [1–4]). Complementary to this approach, ancient DNA data provide direct estimates of past allele frequencies in human populations across time and geography and have refined estimation of the tempo and strength of selection in many instances of selection signals identified in modern genomes. Most recently, population-scale biobank-style datasets, encompassing genomic information and phenotypic data on reproduction, disease, mortality, and other quantitative traits, have pinpointed variants associated with various fitness components, at times in a sex-specific manner. These findings signify the presence of ongoing selection occurring within just one or a few generations.

**Fig 3:**
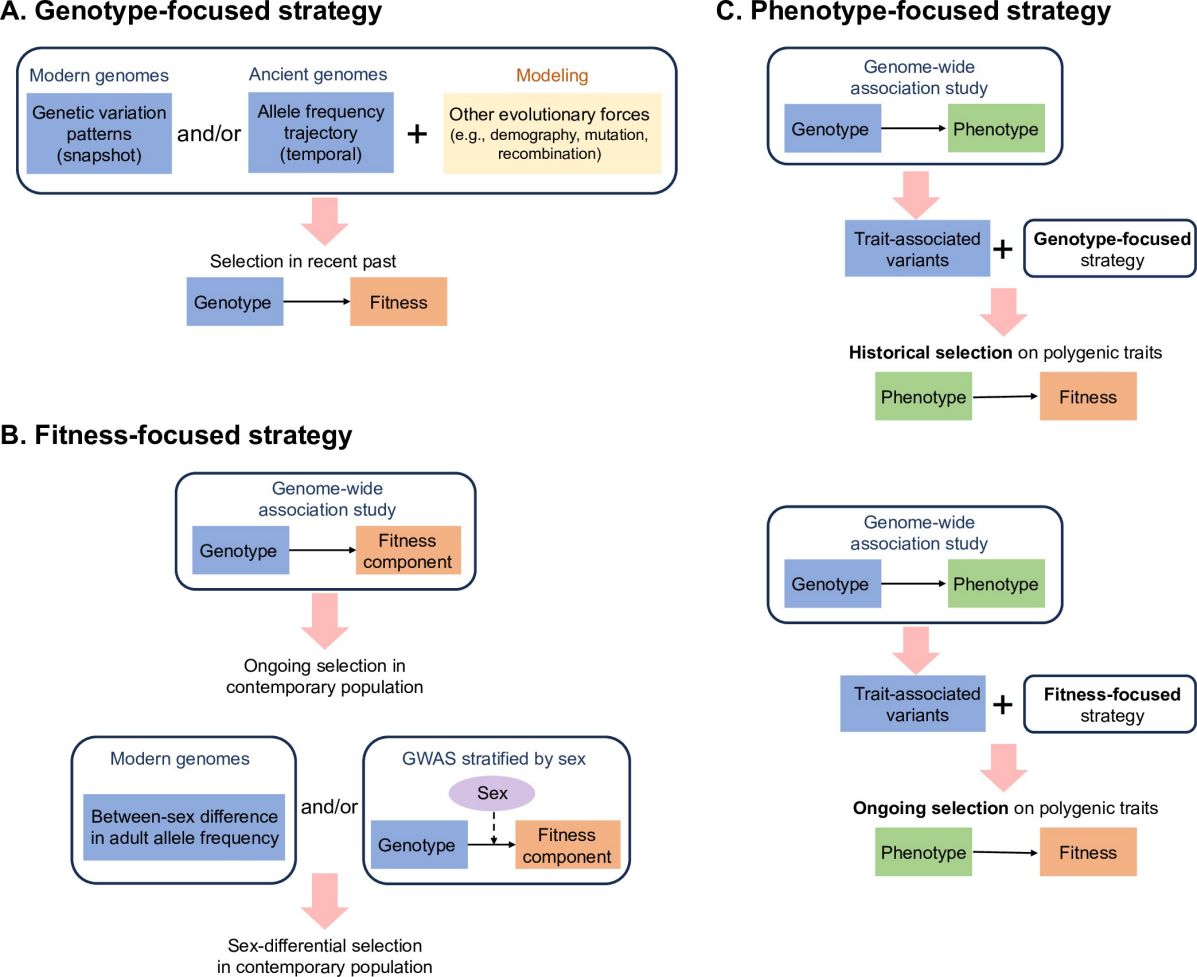
Common strategies for detecting signatures of recent or ongoing selection. (A) A “genotype-focused” strategy focuses on the cumulative effects of historical selection on genetic variation patterns and relies on population genetics modeling to tease apart the influence of other evolutionary forces. Ancient DNA data provide direct information on allele frequency changes, which helps reduce inference uncertainty and confounding by demographic history. (B) A “fitness-focused” strategy focuses on direct association between genotype and fitness component(s) and utilizes allele frequency changes within one generation to detect selection in contemporary populations. As a special case of this strategy, between-sex differences in adult allele frequency or effect size of association to fitness components can be leveraged to detect sex-differential selection. (C) A “phenotype-focused” strategy relies on aggregation of selection signals revealed by genotype-focused or fitness-focused strategies across trait-associated variants identified by genome-wide association studies (GWAS).

This Essay aims to highlight growing evidence for very recent and ongoing genetic adaptation in the human genome, with a focus on positive selection and directional selection on polygenic traits, as these modes of selection may potentially contribute to genetic and phenotypic differences across populations. It is important to note that the effects of negative selection (such as purifying selection and background selection; Box 1) are evident and prevalent in the human genome. However, due to space limitations, this Essay does not discuss the advances made in the past decade in identifying genomic regions and phenotypes subject to recent and ongoing negative and stabilizing selection (e.g., [5–8]). Instead, it only briefly discusses the challenges associated with detecting and interpretating signals of positive and directional selection in the context of pervasive negative selection. The Essay starts with the latest methodological innovations in inference of positive selection at individual genomic loci, and then discusses techniques for detecting aggregate selection signals across genetic loci that collectively influence a quantitative trait. Rather than delving deeply into the technical details, it emphasizes the connection and distinction among “genotype-focused,” “phenotype-focused,” and “fitness-focused” strategies, as well as the advantages and limitations of each (Fig 3). Some major findings stemming from these innovative approaches are discussed, along with challenges in interpretation of the signals.

## Positive selection at individual genomic loci

### Genomic footprints in present-day genetic variation

Traditional methods for detecting selection take a genotype-focused approach (Fig 3A) by adopting classic population genetics models. Specifically, these models predict changes in allele frequency and patterns of surrounding genomic variation by assuming arbitrary fitness effects of different genotypes at a single genetic locus. The obvious advantage of this modeling approach is that it establishes expectations for genomic signatures of selection while requiring very little phenotypic information, such as how genotypes map to phenotypes or which phenotypes are under selective pressure.

Typical genomic signatures of positive selection include extreme differentiation in allele frequencies across populations, extended haplotypes/linkage disequilibrium, or distortion in the site frequency spectrum of segregating variants (reviewed in [9–11]; Fig 2C(i–iii)). These statistics capture complementary features of genomic variation, but most are powerful in detecting selection on intermediate timescales (i.e., hundreds of generations or longer). More recent methods increase detection power by considering multiple summary statistics jointly. This idea was initially implemented using a few basic summary statistics [12] and later expanded through techniques such as Approximate Bayesian Computation [13] or supervised machine learning (reviewed in [14]). Thanks to the recently available population-scale genomic data and continuous theoretical and methodological developments, genome-wide scans based on population genetic summary statistics have identified thousands of putative targets under selection, largely independently of biological knowledge regarding the corresponding phenotype or selective pressure.

Despite being able to pick up selection signals over the past hundreds or thousands of generations, these scans are limited in power for detecting very recent selection because the narrow time window involved leaves very subtle genetic footprints in the site frequency spectrum or haplotype structure. From the perspective of the local genealogical tree, very recent selection only impacts branches near the leaf nodes but leaves most of the tree unchanged. Realizing this, researchers have developed methods that explicitly leverage features of terminal branches of the local genealogical tree. The singleton density score (SDS) is one such method that detects recent allele frequency changes based on extremely rare variants [15]. Specifically, SDS tests for deficiency of singletons (i.e., variants that appear exactly once in the entire sample) on haplotypes carrying the putatively favored allele, which is indicative of a faster coalescent rate in the recent past (Fig 2C(iv)). Along these lines, another method called ascertained sequentially Markovian coalescent (ASMC) detects targets of recent positive selection by inferring pairwise coalescent times and looking for unusually high densities of coalescent events in the recent past (Fig 2C(v)) [16,17]. When applied to whole-genome sequences of approximately 3,200 individuals of European ancestry, SDS detected selection signals in the past 2,000 to 3,000 years in the major histocompatibility complex (MHC) region and at variants associated with lactose tolerance and pigmentation [15]. In comparison, application of ASMC to over 487,000 British individuals identified signals of selection in the past 1,500 years, including those detected by SDS, as well as several new candidate loci harboring genes related to immune response, tumor growth, and other phenotypes [17].

With the recent development of algorithms for inference of the ancestral recombination graph or its proxies, several tree-based statistics have been developed for detecting positive selection (reviewed in [18]; Fig 2C(vi)). One of these methods, Relate, estimates local genealogy from sequence data and detects selection by searching for rapid propagation of lineages carrying a putatively beneficial allele relative to other lineages, effectively testing for differences in the coalescent rate between haplotypes carrying different alleles [19]. However, this selection metric is calculated on only one point estimate of the local genealogy. By contrast, a likelihood method called CLUES leverages the posterior distribution of local genealogical trees to infer selection coefficients and allele frequency trajectories at individual loci [20]. These new methods have confirmed strong selection on variants associated with lactase persistence, immune response, and pigmentation traits in Europeans in the past few thousand years and some signals in other populations (such as the *EDAR* gene in East Asians), although very few new signals have been detected.

### Selection signals in ancient genomes

While modern genomes provide a snapshot of population evolution and allow for indirect inference of past demographic and selective events, genomic sequences from ancient samples enable direct glimpses into the genetic history of human populations. By providing estimates of allele frequencies at multiple time points (Fig 2A and 2B), ancient DNA has shed valuable insights on the evolutionary histories of multiple selected variants in human evolution during the past 15,000 years (reviewed in [21–23]). Analysis based on ancient DNA has also been particularly helpful in detecting candidates under spatially or temporally restricted selection.

Ancient DNA transformed our understanding of selection in humans by resolving complex interactions between selection and demographic history. As recent human history features many episodes of population splits and admixture, signals of selection are often obscured by changes in ancestry [24]. One instance is the evolutionary history of the *FADS* locus, which contains genes encoding enzymes involved in the conversion of long-chain polyunsaturated fatty acids. Using present-day genomic data, studies detected strong selection signals on *FADS* genes in human populations from multiple continents, with different alleles being favored across time and geography [25–29]. However, analysis of ancient DNA showed that the selection signal in Native Americans was largely an artifact driven by parallel selection in European and Asian populations [30]. Another intriguing case is the evolution of pigmentation in west Eurasia in the context of several major admixture events revealed by ancient DNA. The derived alleles associated with lighter skin or eye color at several pigmentation-associated genes exhibited distinct frequencies in different ancestral populations, potentially reflecting differential selective pressures across geography prior to the Mesolithic period (i.e., before 9,000 to 10,000 years ago) [31,32]. Moreover, the observed allele frequencies and ancestry fractions at these pigmentation-associated variants in later admixed populations significantly deviated from neutral expectations, suggesting subsequent selection during the Neolithic, Bronze Age, and historical periods [33–35]. These findings point to continued selective pressure for light pigmentation over the past 2,000 years in west Eurasia and support the concept that admixture may facilitate rapid adaptation by introducing advantageous alleles [34–37].

Ancient DNA data have also refined our knowledge of the onset, duration, and strength of selection events. For example, selection on the variant conferring lactase persistence was initially estimated to begin around 7,500 years ago based on modern genomic data and archeological evidence of dairy production [38]. Surprisingly, ancient DNA data have shown that the selected allele was rare in Bronze Age Europe until 3,000 years ago, suggesting a much later onset of positive selection than was previously inferred [31]. In addition, based on the allele frequency trajectory in ancient DNA samples, the positive selection for this allele was inferred to be strong 100 to 150 generations ago but drastically reduced in the past 100 generations [39]. Significant variation in selection strength has also been found at several other previously identified selected loci [39]. Overall, ancient DNA studies have confirmed selection signals near multiple genes associated with diet, pigmentation, and immune response revealed in modern genomic data, and have provided fine-resolution insights into the temporal dynamics and geographic distribution of the selected variants and the corresponding selection strengths [26,34,39,40].

With recurrent observations of selection targeting genes in immune pathways, the quest to discern the specific pathogens driving these selective pressures has been immensely captivating. A strategy to link selection signals with the causative pathogens is to search for variants with unusual allele frequency changes during well-documented catastrophic pandemics. A recent investigation scrutinized ancient genomes of roughly 200 individuals who died before, during, and after the Black Death pandemic in the fourteenth century [41]. This study reported an overall enrichment of allele frequency differentiation in immune genes as well as a handful of potential targets under positive selection. However, serious skepticism has been raised towards the findings due to technical concerns [42], and other studies adopting similar designs (though with smaller sample sizes) failed to replicate the selection signals at immune genes overall or at individual candidates [43,44]. These results suggest the selection effects of historical pandemics at individual genomic loci are relatively modest, necessitating expansive sample sizes for detection.

### Fitness-focused strategy for detecting selection in contemporary populations

The fitness of an individual consists of several components such as viability, mating success, and fecundity. A genetic variant that influences any of these components is subject to natural selection unless its effects on all components cancel out. Based on this reasoning, one can identify loci under ongoing selection using a fitness-focused approach by performing GWAS on proxies for fitness components (Fig 3B). However, traits closely associated with fitness are expected to have low heritability [45], and fitness-related variants tend to be rare in frequency. Therefore, identification of these variants via association requires exceedingly large sample sizes, which only became feasible in the past decade. It is worth noting that, due to limited power, this association approach is biased towards detecting common variants and does not pick up fitness-influencing variants that are under strong negative selection.

One of the most studied proxies of fertility is the number of children ever born to or fathered by an individual, because it can be easily surveyed and approximates the overall fitness well in modern populations with low mortality. Using data from hundreds of thousands of individuals born in the 1950s to 1970s, dozens of genomic loci have been associated with the number of children [46–48]. Interestingly, among the top associations stands the *FADS* locus, which also harbors strong signals of historical positive selection in both ancient and present-day DNA samples [26,28,29,49]. By contrast, the two most significant association regions lack evidence of historical positive selection but demonstrate signals of balancing selection, possibly due to pleiotropic effects (Box 1) on other fitness components or temporally fluctuating selection [46,50,51].

Besides reproduction, viability is a key component of fitness. In principle, the number of children closely reflects their contribution to the population gene pool of the next generation, but current association studies for this trait include only individuals who survived to completion of their reproductive lifespan, leaving out those who did not reach adulthood. To detect common variants linked to early-life survival, Wu and colleagues performed a clever GWAS on time- and location-matched infant mortality rate (IMR) for living individuals in the UK Biobank [52]. The rationale is that individuals who survived in tougher environments during infancy, as indexed by a higher local IMR in their birth years, tend have higher “relative viability.” Interestingly, the two genome-wide significant loci identified by this approach, *LCT* and *TLR6-TLR1-TLR10*, are both known targets of recent positive selection in Europeans, with the survival-increasing alleles matching the evolutionarily favored allele [15,26].

A more direct approach for identifying variants that affect viability is by looking for shifts in allele frequency across individuals of different ages [2]. Limited by the age distribution of participating individuals in current cohorts, this method is underpowered to detect allele frequency changes in early life, when selective pressure is expected to be strong. However, in humans, even variants that exclusively affect viability late in life may be under selection, due to late male reproduction, intergenerational resource transfer, and other reasons [53,54]. By testing for changes in allele frequency with age, a study found and replicated two genome-wide significant signals in 2 independent datasets: one overlaps with the *APOE ε4* allele that is associated with reduced lifespan and increased risk of Alzheimer’s disease and cardiovascular diseases [55,56]; the other locus contains variants that are close to a nicotine receptor gene *CHRNA3* and associated with increased smoking quantity [57]. Intriguingly, the relatively common frequencies of these survival-reducing variants in present-day populations suggest that they were not under strong negative selection in the recent past. The authors interpreted the lack of abundant associations as evidence for purifying selection against variants with large effects on late-onset disease and speculated that the *APOE* and *CHRNA3* loci were found because their deleterious effects have recently increased in humans due to environmental changes.

### Fitness-focused strategy for detecting sex-differential selection

The extraordinary level of sexual dimorphism in many animal species, including humans, reflects sex-specific phenotypic effects and sex differences in the fitness landscape. The fitness effect of a genetic variant may differ between sexes in magnitude or sometimes in direction. Such sex-differential selection is challenging to study because mendelian inheritance equalizes autosomal allele frequencies between the 2 sexes at fertilization in each generation. Nevertheless, the special case of sex-differential selection on viability is expected to leave a distinctive signature in population genetic variation: allele frequency differences between adult females and males (Fig 3B, right). An early study seeking this signature reported signals at hundreds of genetic regions and an enrichment of signals on the X chromosome compared to autosomes [58]. Unfortunately, these findings turned out to be largely false positives driven by random noise, sex-biased genotyping error, and biases due to hemizygosity of the X chromosome in males. Later studies on much larger biobank datasets failed to detect robust signals at any autosomal loci [59] or enrichment on the X chromosome [60].

While signals of sex-differential viability selection are expected to be exceptionally weak at individual loci [61,62], subtle between-sex allele frequency differences across many variants may be detectable in aggregation. Leveraging the genomic and reproductive history data of approximately 250,000 adults in the UK Biobank, Ruzicka and colleagues developed new metrics to measure between-sex allele frequency differentiation over different stages of a life cycle. They found significant shifts in the genome-wide distributions of these metrics, which is consistent with effects of sex-differential selection on survival, reproductive success, and overall fitness [4].

### Limitations of the fitness-focused strategy for selection detection

One curious observation from the studies described above is the limited overlap between fitness-associated variants in contemporary populations and targets under historical positive selection. As the 2 approaches (i.e., fitness-focused and genotype-focused) capture selection signals of very different timescales, one explanation is a highly dynamic selection landscape during recent human evolution. However, the fitness-associated variants identified in biobank-style datasets need to be taken with a grain of salt for several technical reasons.

First, the effect measured by association likely does not reflect the actual fitness effect. Fitness effects that are “visible” to natural selection may be too subtle to be picked up by association studies given current sample sizes, so many targets of ongoing selection might be missed. On the other hand, proxy traits only capture certain aspects of fitness components, so the measured effect of a variant may be greater than its effect on overall fitness in the presence of antagonistic pleiotropy (Box 1). In other words, there may be weaker or even no ongoing positive selection on variants with opposite effects on different fitness components.

Second, as for all GWAS in general, uncorrected population stratification remains a concern for fitness-associated variants, especially for those with highly differentiated frequencies across populations. For example, the lactase persistence variant near *LCT*, the top selection target identified by the IMR GWAS, is among the most differentiated variants across European populations [63]. Despite the authors’ best effort in correcting for population structure, it is still possible that the IMR association signal in UK Biobank data is driven by residual stratification, so the claim of ongoing selection on this variant remains to be validated in independent datasets or by family-based approaches [64].

A related yet different issue applies to analysis based on allele frequency differences between sex. In addition to sex-biased viability selection, between-sex allele frequency differences can also be interpreted as the result of subtly different population structures between sexes or sex-biased participation [65]. The UK Biobank requires active participation, and the participants are not representative of the general population in various sociodemographic and health-related characteristics [66]. Should a genetic variant affect participation inclination in men and women differentially, subtle allele frequency difference between sexes is expected. Consistent with this hypothesis, a “GWAS of sex” performed in 5 biobank-style datasets found significant positive autosomal single-nucleotide polymorphism heritability in those that require active participation (including UK Biobank) but not those with relatively passive recruitment, although this contrast is confounded by differences in sample size across datasets [65]. Therefore, an important future step will be to replicate the findings in more population-representative datasets or family-based studies to rule out or quantify the contribution of sex-differential participation bias.

## Directional selection on quantitative traits

### Integration of GWAS results with genetic variation patterns

GWAS have provided unprecedented insights into the genetic architecture of human phenotypes, revealing significant heritability and high polygenicity (Box 1) of most traits, as well as unexpectedly small effect sizes for most associated variants. These observations are surprisingly close to the assumptions of classical quantitative genetics models [67]. In the context of adaptation, the measurable heritability means that at least a portion of the phenotypic variation within a population is attributed to existing genetic polymorphisms, which, in response to changes in selective pressure on the phenotype, offer the materials for genetic adaptation without having to await new mutations. In turn, the high polygenicity and tiny effect sizes of most variants suggest that the selective pressure on any individual alleles may be too small to leave discernible genomic footprints but may be detectable in aggregate. These considerations point to the importance of examining polygenic signals of selection on traits during human evolution via a phenotype-focused strategy (Fig 3C) [68].

If all or most trait-influencing variants can be identified in an unbiased manner, signals at these loci can be interrogated jointly to uncover selection on the trait. The most straightforward idea for detecting polygenic adaptation is to directly combine GWAS results and population genetic summary statistics (e.g., some in Fig 2C) [15,19,33–35,69]. Common approaches include tests for shifts in distribution of single-locus summary statistics indicative of selection (e.g., *F*_*st*_) at GWAS hits [69] or correlation between GWAS summary statistics (such as effect direction, magnitude, and significance level) and population genetic summary statistics. This approach has been applied to both present-day and ancient DNA data, and several studies explicitly leveraged population admixture events in recent human history to gain insights into the timing of selection [15,19,33–35,70]. Overall, these studies found consistent evidence of selection on variants underlying anthropometric, pigmentation, and immune-related trait variation in human populations in the past 10,000 years.

Rooted in the classic quantitative genetics model, more direct tests for polygenic adaptation have been devised around the concept of “genotypic value” (also known as the “breeding value” in quantitative genetics when nonadditive genetic effects are ignored) that describes the total contribution of all genetic variants of an individual to their phenotypic value. The polygenic score (PGS)—the sum of allele effect sizes across all independent GWAS loci—provides a proxy for the genotypic value that can be applied at the individual or population level. In addition to empirical comparison of observed PGS to a null distribution based on sets of matching variants [71,72], formal tests for polygenic signals of selection on quantitative traits have been developed in the population genetics framework [73,74]. In a way, these tests are analogous to tests for single-locus selection, but instead of rapid change or differentiation of allele frequency, signals for polygenic adaption come from unexpected changes or overdispersion of PGS in the history of one or multiple populations [73,74].

GWAS results have also been explicitly incorporated into the coalescent framework. By combining GWAS effect sizes and inferred local genealogical tree at GWAS loci, Edge and Coop developed methods for reconstructing the trajectory of population-mean PGS over time [75]. They applied these methods to test polygenic signals of selection for increased height in the British population but only found very weak signals concordant with prior reports [15,70,73,74]. Taking a different approach, Stern and colleagues extended their method CLUES to estimate selection intensity on a polygenic trait by considering the allele frequency trajectories of GWAS loci conditional on the inferred local coalescent trees [76]. Contrary to the conclusion of prior studies, this method detected no signal of recent directional selection on height or body mass index, but replicated some other traits previously reported to be under recent selection, such as pigmentation traits, age at first birth, glycated hemoglobin, and educational attainment. By combining theory of quantitative genetics and population genetics and incorporating empirical GWAS findings, these new methods unveiled many signals of selection on quantitative traits during recent human evolution and are paving the way for many more future findings.

### Correlation between phenotypes and fitness components

Analogous to the fitness-focused approach for detecting ongoing selection at individual loci, selection effects on a polygenic trait can be estimated based on phenotypic or genetic correlations between the trait and a proxy of fitness component [77]. Approaches include regression of a measure of reproductive success on PGSs for traits of interest [78,79] or estimation of genetic correlation between traits of interest and proxies for fitness [80,81]. Partially consistent with previous epidemiological studies, these studies found selection in contemporary human populations for genetic variants underlying earlier age at first birth and shorter stature in females, as well as for those underlying increased body mass index and reduced educational attainment in both sexes.

Hypothesizing that variants influencing polygenic traits may be under sexually antagonistic selection on viability, Zhu and colleagues developed a test based on between-sex allele frequency differences and sex-specific phenotypic effect sizes from GWAS. They found suggestive signals of selection on testosterone levels [82], which is consistent with the recent findings of positive correlation between testosterone level and mortality in females and an inverse relationship in males [83]. Nonetheless, because the model makes some strong assumptions, such as allele frequency under equilibrium and selection coefficient proportional to phenotypic effect size, it remains questionable whether the detected signal is specific to sexually antagonistic selection or can also reflect the effects of other evolutionary processes.

### Challenges in validating and interpreting polygenic signals of selection on quantitative traits

Despite significant progress in detecting polygenic adaptation in the past decade, serious concerns quickly emerged regarding the validity and interpretation of the reported signals of polygenic adaptation, for both technical and conceptual reasons [84]. First, technical biases in GWAS may lead to false positive signals or biased effect size estimates at individual loci. For example, the strong signals of selection on height in Europeans were found to largely result from uncorrected population stratification and weakened considerably with effect size estimates from GWAS of less-structured samples [75,85,86]. The inherent ascertainment bias and limited portability of GWAS results cast additional uncertainty on the reliability of selection signals when applying GWAS summary statistics from a study group to selection tests in other groups [84,87,88]. Furthermore, although being intuitive and powerful for combining information across sites, PGSs, especially those constructed with variants that do not reach genome-wide significance, further exacerbate biases of GWAS results due to residual population stratification [89].

Moreover, most current methods fundamentally test for deviation from neutrality (i.e., no selection on any trait or variant at all), so the detected signals may reflect effects of other modes of selection. Despite the debate on the prevalence of polygenic adaptation, there is a consensus that GWAS variants with large effect sizes are under negative selection, indicated by the strong negative correlation between variant effect size and minor allele frequency (beyond the expectation under detection bias) [90–92]. This phenomenon is consistent with the action of stabilizing selection (Box 1) on quantitative traits: For a population centered around the phenotypic optimum, mutations that affect fitness-relevant phenotypes tend to shift the population away from the optimum and thus be deleterious [93]. The prevalence of stabilizing selection leads to challenges in detection and interpretation of population differences in PGS. First, under stabilizing selection, adaptive genetic changes do not always mirror shifts in the phenotypic optimum. Environmental changes can alter not only the optimal trait level (Fig 1; dashed purple arrow) but also the mean environmental contribution to the phenotype (Fig 1; solid purple arrow), which induces “genetic compensation” in the opposite direction [84,94]. Second, although stabilizing selection around the same trait optimum constrains phenotypic differentiation between populations, it accelerates genetic differentiation at trait-influencing loci. This counterintuitive effect of stabilizing selection, combined with incomplete and biased ascertainment of GWAS loci, inflates differences in PGS between populations and may even yield spurious signals of polygenic adaptation [95]. These considerations underscore the importance of regarding stabilizing selection (with a constant trait optimum throughout time and space) as a null model for devising and interpreting tests for polygenic adaptation, especially those reliant on inter-population comparisons.

Even when the selection signals are technically sound and effects of stabilizing selection are adequately considered, it remains a formidable challenge to tell which traits are directly under selection, given the prevalent pleiotropy (Box 1) across human complex traits [96–98]. Aware of this issue, researchers developed methods that aim to disentangle effects of selection on genetically correlated traits and found evidence of indirect selection (e.g., signals of selection on educational attainment due to selection on other traits) and opposing selection (e.g., selection for increased type 2 diabetes and decreased glycated hemoglobin), which helps with the rejection of the hypothesis that a certain trait is under direct selection [76]. Yet, this study only tested for correlated response of 137 pairs of traits and may have missed signals driven by multi-way pleiotropy or unmeasured traits [96]. In other words, given current data and methods, one can at best conclude selection on variants associated with certain trait(s) but not selection on the trait(s).

## Conclusion and future directions

Rapid growth in genomic datasets and advances in computational techniques have enabled identification of parts of the human genome under very recent or ongoing adaptive selection. Early genome-wide selection scans relied on the cumulative effects of selection over relatively long timescales, but statistical innovations have enabled efficient computation using large numbers of modern genomes to study selection over narrower time frames. The utilization of ancient DNA data has further reduced inference uncertainty and confounding due to demographic history, providing valuable insights into the temporal dynamics and geographic distribution of the selected variants. We now have lists of candidate targets with compelling evidence of selection during the past 15,000 years, along with partial information regarding variation in the selection strength.

As selection on genotypes is mediated by differences in fitness-relevant phenotypes (Fig 1), a complete understanding of selective events involves not only the causal variants but also the relevant phenotypes and selective forces [99]. Integrating rich phenotypic data with genomic information in population-scale datasets has facilitated the establishment of associations between variants and phenotypes. Following numerous association studies conducted for both organismal and molecular phenotypes, it is increasingly clear that pleiotropy is widespread across human traits [97,98,100]. It is possible that many inferred selective variants will be associated with multiple phenotypes in future GWAS, so the new questions will become: which of these phenotypes, if any, is mediating selection; where does the selection pressure come from; and is selection still ongoing in present-day populations?

The expanding biobank datasets will be pivotal in addressing these questions. First, they offer an opportunity to directly identify individual or groups of variants associated with fitness components. The partial overlap between fitness-associated variants and those targeted by historical positive selection may arise from limited power to detect subtle fitness effects, antagonistic effects on different fitness components (and/or between sex), or spatial or temporal variation in fitness effects. With the anticipation of long-term longitudinal data, possibly spanning from birth to death, becoming available in the next few decades, it will be possible to develop new statistics that better approximate various fitness components and integrate them throughout a complete life cycle, thus enhancing power to identify variants that influence the overall fitness. It is important to note that since such discoveries are associations in nature, replication in additional biobank datasets or by family-based studies will be crucial.

Second, the rich phenotype data, coupled with theoretical advancements, can potentially distinguish between traits directly or indirectly under selection. Although it remains uncertain which and how many pleiotropically related traits collectively shape the fitness landscape, emerging evidence suggests that, at least for some traits, a model featuring many traits under stabilizing selection aligns well the empirical GWAS results [3]. These considerations strongly advocate for incorporating pleiotropy alongside stabilizing selection in future models and simulations that characterize genetic signatures of polygenic adaptation [101,102]. Findings from such models, combined with variant-level pleiotropic effect size estimates from empirical association studies, may unveil clearer adaptation signals and help differentiate between traits directly or indirectly influenced by selection.

Lastly, given the emerging evidence of sex differences in phenotypic and fitness effects of the same variant [4,82], along with varying prediction accuracy of PGSs across different contexts (e.g., age, sex, income level) [88], more context-dependent effects will likely be unmasked. These findings may imply gene-by-environment interactions on phenotype and fitness, hinting at the environmental conditions that exert selective pressure. This information, when combined with archeological data about past environments, diets, and lifestyles of human populations, may aid in rejecting and formulating new hypotheses regarding recent selective forces that have shaped the human genomic and phenotypic variation.

## Abbreviations

ASMC: ascertained sequentially Markovian coalescent
GWAS: genome-wide association study
IMR: infant mortality rate
MHC: major histocompatibility complex
PGS: polygenic score
SDS: singleton density score
